# Prognostic significance of circulating tumor cell measurement in the peripheral blood of patients with nasopharyngeal carcinoma

**DOI:** 10.1016/j.clinsp.2023.100179

**Published:** 2023-03-23

**Authors:** Tinghua Gao, Jinxing Mao, Jindu Huang, Fengling Luo, Lixiang Lin, Yingni Lian, Sanmei Bin, Lianghua Zhao, Shuping Li

**Affiliations:** Department of Oncology, First People's Hospital of Zhaoqing City, Zhaoqing, Guangdong, China

**Keywords:** Nasopharyngeal carcinoma, Circulating tumor cells, Clinical pathological features, Treatment

## Abstract

**Objective:**

Nasopharyngeal Carcinoma (NPC) is lethal cancer. Typically, relapse and metastasis are the outcomes of most patients. Against this backdrop, this study aimed to investigate the correlation between Circulating Tumor Cell (CTC) profiles and clinicopathological features in patients with NPC.

**Patients and methods:**

A total of 119 blood samples from 79 patients were collected from patients with NPC during treatment. CanPatrol^TM^ CTC enrichment and RNA In Situ Hybridization (RNA-ISH) were used to characterize CTCs, including epithelial, Mesenchymal (MCTCs), and epithelial/mesenchymal mixed types according to their surface markers.

**Results:**

The number of CTCs and MCTCs in the pre-treatment group was significantly higher than that in the post-treatment group (p < 0.05). The total number of CTCs and MCTCs cell numbers was significant correlation with Tumor-Node-Metastasis (TNM) staging (p < 0.05), Progression-Free Survival (PFS), and Overall Survival (OS). The PFS of patients with > 7 CTCs or > 5 MCTCs per 5 mL blood was significantly shorter PFS than those patients with ≤ 7 CTCs or ≤ 5 MCTCs (p < 0.05). Patients treated with targeted therapy combined with chemoradiotherapy had poorer PFS and OS rates than those treated with chemoradiotherapy (p < 0.05). The Kaplan-Meier survival analysis also demonstrated that patients with changes in CTC > 4 were strongly associated with PFS and OS rates (p < 0.05).

**Conclusion:**

CTC and MCTC number detection in patients with NPC is a useful biomarker for predicting patient progress. Patients with more than 7 CTCs or 5 MCTCs in 5 mL of blood had shorter PFS and OS rates. CTC and MCTC count changes were also significantly associated with the patient's therapy.

## Highlights

• The number of total CTCs and MCTCs had a strong correlation with Tumor-Node-Metastasis (TNM) stages (p < 0.05), Progression-Free Survival (PFS), and Overall Survival (OS).
• The patients with > 7 CTCs or > 5 MCTCs per 5 mL blood had poorer PFS.
• The PFS of the patients with chemotherapy combined targeted therapy had shorter PFS than that of the patients with chemoradiotherapy.
• The patients with > 4 CTC counts change between pre-treatment and post-treatment had shorter PFS survival rates and overall rates (p < 0.05).

## Introduction

Nasopharyngeal Carcinoma (NPC) is one of the most lethal cancers worldwide.^1^ In China, NPC has a very high incidence in the southeastern mainland.^2^^,^^3^ The occurrence of NPC is frequently associated with virus infections, environmental factors, and heredity.^4^ Epstein-Barr virus infection is often associated with NPC.^5^ NPC treatments include surgery, chemotherapy, and radiotherapy.^6^ Sometimes, combined chemotherapy and radiotherapy are required.

Although the treatment of NPC has progressed greatly in the past few decades, NPC relapse eventually occurs. Reportedly, the presence of Circulating Tumor Cells (CTCs) in the peripheral blood of patients with NPC is frequently associated with cancer relapse.^7^^,^^8^ Moreover, relapse and metastasis in patients with positive CTCs are strongly related to the Epithelial-Mesenchymal Transition (EMT) mechanism. A few critical molecules are involved in the EMT process, including E-cadherin,^9^ Cytokeratins (CK), N-cadherin, fibronectin,vimentin, Twist, and Snail.^10^, ^11^, ^12^, ^13^ Many studies show that Epithelial Cell Adhesion Molecule (EpCAM) and CK8/18/19 are epithelial cell markers. In contrast, Twist 1 and vimentin are specific markers for mesenchymal cells.^13^^,^^14^ NPC mainly occurs in China and Southeast Asia and is a common malignant tumor of the head and neck.^15^^,^^16^ With increasing improvements in radiotherapy techniques, the 5-year Overall Survival (OS) rate of patients with NPC has reached 70% in China.^2^ However, 30%–40% of patients eventually develop recurrence or distant metastasis. Therefore, the management of patients with relapse and metastasis is a major challenge. Emerging evidence indicates the requirement for a more reliable and sensitive method for detecting rare NPCs after treatment.

Recent studies have revealed that CTCs originate from primary tumors and can enter the circulation system.^17^^,^^18^ This mechanism may be a major reason for the recurrence and metastasis of many malignant tumors in the lung, stomach, breast, head and neck.^19^, ^20^, ^21^, ^22^, ^23^, ^24^ Additionally, with advances in liquid biopsies, CTC-based detection can not only play a key role in monitoring NPC clinical progress but also guide the treatment of patients. Therefore, changes in CTC count in patients with cancer have great clinical value in assessing their diagnosis, therapy, relapse, metastasis,and prognosis. A recent study also revealed that changes in CTC count during treatment were closely associated with the effects of NPC therapy. Therefore, if CTCs are continuously monitored in a timely manner, the prognosis of patients with NPC will be clearly recorded. Here, the authors investigated the relationship between CTC profiles and outcomes of patients with NPC.

## Materials and methods

### Subjects and samples

This study enrolled 79 patients with NPC, including 53 men and 26 women, aged 23 to 79 years. Patients were diagnosed and admitted to the present hospital between November 2014 and October 2020. Following the Tumor-Node-Metastasis (TNM) staging system for NPC in the 2008 version, the number of patients with stages I, II, III, Iva and IVb were 2, 6, 33, 27 and 11, respectively, and the diagnosis was confirmed by histopathological specialists based on tumor biopsy or fine-needle aspiration cytology. TNM staging was determined by combining clinical characteristics and pathological types. The following conditions were excluded from this study: active infection and malfunction or failure of key organs such as the liver, kidney, and heart. A total of 119 peripheral blood samples from 79 patients with NPC, including 70 samples in pre-treatment and 49 samples in post-treatment groups, were collected, and CTCs were identified via CanPatrol^TM^ CTC enrichment and RNA In Situ Hybridization (RNA-ISH) techniques before or after treatment because this technique has very sensitivity and specificity.^14^^,^^25^

The study was reviewed and approved by the ethics committee of the First People's Hospital of Zhaoqing City (approval number #:2018-11-02). Written informed consent was obtained from all the patients before the study. All procedures performed in studies were in accordance with the ethical standards of the 1964 Declaration of Helsinki and its later amendments or comparable ethical standards.

### Processing of CTCs

The authors used a filtration method to identify the CTCs as in previous descriptions.^25^^,^^26^ Briefly, all blood samples were processed in a filtration tube system (SurExam, Guangzhou, China) in the laboratory, including a calibrated membrane with 8-μm diameter pores (Millipore, Billerica, USA), a manifold vacuum plate with valve (SurExam, Guangzhou, China), and a vacuum pump (Auto Science). First, a total of 5 milliliters (mL) of peripheral blood from patients was taken in veins 1‒3 days at pre-treatment and post-treatment and loaded into Ethylenediaminetetraacetic Acid (EDTA) coated tube. Then, erythrocytes in the collected blood sample were removed using lysis buffer (Sigma, St. Louis, USA, Cat#:R7757‒100 mL). The remaining cells were centrifuged and discarded into the supernatant. The cell pellet was suspended and fixed in 4% formaldehyde in Phosphate-Buffered Saline (PBS) for 5 minutes. Finally, the cell suspension was connected to the filtration tube in vacuum conditions, and samples were processed.

### Characterization of CTCs via Tri-color RNA in situ hybridization

To identify CTCs, the authors used the RNA-ISH technique for this study, which is based on branched DNA (bDNA) signal amplification technology. Signals of biomarkers of interest were amplified based on the binding of a bDNA capture probe to the target sequences.^27^ Briefly, the targeted DNA sequences were first bound by multiple bDNA capture probes; bDNA signal amplification probes were then bound to bDNA capture probes. Finally, the bDNA amplifier probe with different fluorescent dye labeling was identified using an immunofluorescence microscope (Olympus, Tokyo, Japan). The capture probes for EpCAM and CK8/18/19 specifically bound to epithelial markers were labeled with Alexa Fluor 594 showing red color. Vimentin and twist were specific mesenchymal markers and labeled with Alexa Fluor 488 showing green color. The capture probes were purchased from SurExam Company (SurExam, Guangzhou, China) and the sequences are in Table 1. The authors also performed 40,6-Diamidino-2-Phenylindole (DAPI) staining to gate nuclei shape. All process was performed in 24-well plates (Corning, USA) and carried out at 40°C.

**Table 1:** Capture probe sequences for the EpCAM, CK8/18/19, vimentin, and Twist.

Gene name	Sequences (5´-3´)
EpCAM	5´-TGGTGCTCGTTGATGAGTCA
	AGCCAGCTTTGAGCAAATGA-3´
CK8	5´-CGTACCTTGTCTATGAAGGA
	ACTTGGTCTCCAGCATCTTG-3´
CK18	5´-AGAAAGGACAGGACTCAGGC
	GAGTGGTGAAGCTCATGCTG-3´
CK19	5´-CTGTAGGAAGTCATGGCGAG
	AAGTCATCTGCAGCCAGACG-3´
Vimentin	5´-GAGCGAGAGTGGCAGAGGAC
	CTTTGTCGTTGGTTAGCTGG-3´
Twist	5´-ACAATGACATCTAGGTCTCC
	CTGGTAGAGGAAGTCGATGT-3´

### Statistical analysis

All data analyses were performed via statistic software SPSS 24.0 (IBM Inc, Chicago, IL, USA). CTCs positive rate and comparison among different groups were implied via the χ2 test, *t*-test, and Kruskal-Wallis test. For two-group comparisons, the authors employed the Mann-Whitney *U* test. Fisher's exact and Pearson Chi-Square tests were used to analyze relationships between CTCs number and clinical characteristics including age, gender, smoking, and TNM staging. PFS and OS rates were calculated via Kaplan-Meier analysis; p < 0.05 was considered statistically significant.

## Results

### Patients and CTC characteristics

**Figure 1:**
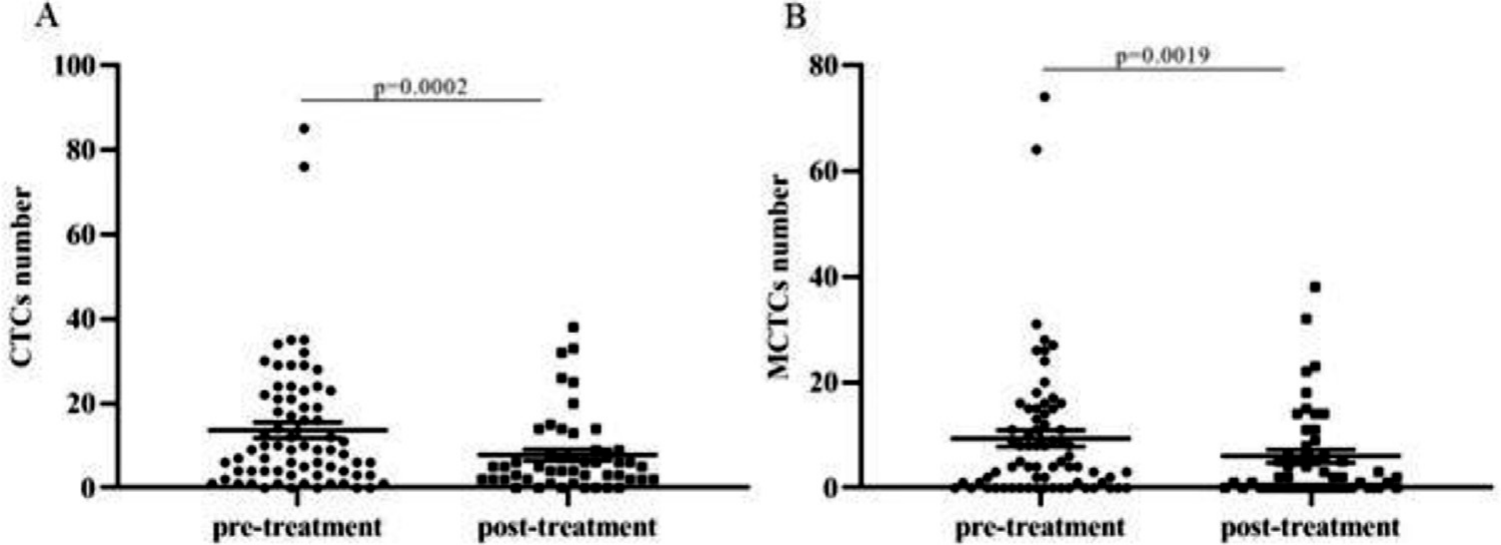
**Correlation between CTCs or MCTCs count and treatment.** (A) The comparison of CTCs count between pre-treatment and post-treatment. (B) The comparison of MCTCs count between pre-treatment and post-treatment. CTC, circulating tumor cell; MCTC, mesenchymal circulating cell.

A total of 79 patients were recruited in this study, and their detailed characteristics are shown in Tables 2 and 3. A total of 119 blood samples were collected during treatment. Based on the collection time of blood samples, the patients were divided into pre- and post-treatment groups. In the pre-treatment group, the positive CTCs were counted in 65 of 70 patients (92.9%) with a median number of 5.00 CTCs in 5 mL blood (range 0‒62). In the post-treatment group, positive CTCs were identified in 42 of the 49 patients (85.7%) with a median number of 4.00 CTCs in 5 mL blood (range 0‒51). CTCs were categorized into three phenotypes according to their surface markers: epithelial, biphenotypic, and Mesenchymal CTCs (MCTCs). Similarly, positive MCTCs were counted in 52 of the 70 patients (74.3%) with a median number of 4.00 CTCs in 5 mL blood (range 0‒61) in the pre-treatment group and in 33 of the 49 patients (67.3%) with a median number of 3.00 CTCs in 5 mL blood (range 0‒44) in the post-treatment group (Tables 2 and 3). Additionally, there were significant differences in the number of CTCs and MCTCs between the pre-treatment and post-treatment groups (Fig. 1 A and B). These data indicated that CTCs and MCTCs both decreased after treatment.

**Table 2:** The detailed characteristics of 70 blood samples collected before treatments (Fisher's exact test and Pearson chi-square test).

Variables	n (%)	CTCs		p	MCTCs		p
		Positive, n (%)	Negative, n (%)		Positive, n (%)	Negative, n (%)	
All samples	70	65 (92.86)	5 (7.14)		52 (74.29)	18 (25.71)	
Age (years)							
Mean	52.00						
SD	10.123						
Range	23‒79						
Gender				0.724			0.225
Male	47 (67.14)	44 (93.62)	3 (6.38)		37 (78.78)	10 (21.28)	
Female	23 (32.86)	21 (91.30)	2 (8.70)		15 (65.22)	8 (34.78)	
Smoking				0.362			0.066
No	32 (45.71)	30 (93.75)	2 (6.25)		27 (84.38)	5 (15.63)	
Yes	38 (54.29)	35 (92.11)	3 (7.89)		25 (65.79)	13 (34.21)	
TNM stage				0.798			0.520
I	1 (1.43)	1 (100.00)	0 (0.00)		0 (0.00)	1 (100.00)	
II	6 (8.57)	5 (83.33)	1 (16.67)		5 (83.33)	1 (16.67)	
III	28 (40.00)	27 (96.43)	1 (3.57)		21 (75.00)	7 (25.00)	
IVa	26 (37.14)	24 (92.31)	2 (7.69)		19 (73.08)	7 (26.92)	
IVb	9 (12.86)	8 (88.89)	1 (11.11)		7 (77.78)	2 (22.22)	
Distant metastasis				0.797			0.620
Yes	9 (12.86)	7 (77.78)	2 (22.22)		8 (88.89)	1 (11.11)	
No	61 (87.14)	45 (73.77)	16 (26.23)		57 (93.44)	4 (6.56)	
Lymph node metastasis				0.623			0.097
Yes	67 (95.71)	62 (92.54)	5 (7.46)		51(76.12)	16 (23.88)	
No	3 (4.29)	3 (100.00)	0 (0.00)		1 (33.33)	2 (66.67)	

CTCs, Circulating Tumor Cells; n, Case Number; MCTCs, Mesenchymal and Mixed CTCs; SD, Standard Deviation; TNM, Tumor-Node-Metastasis.

**Table 3:** The detailed characteristics of 49 blood samples collected after treatments (Fisher's exact test and Pearson Chi-Square test).

Variables	n (%)	CTCs		p	MCTCs		p
		Positive, n (%)	Negative, n (%)		Positive, n (%)	Negative, n (%)	
All samples	49	42 (85.71)	7 (14.29)		33 (67.35)	16 (32.65)	
Age (years)							
Mean	50.00						
SD	9.112						
Range	35‒69						
Gender				0.136			0.071
Male	33 (67.35)	30 (90.91)	3 (9.09)		25 (75.76)	8 (24.24)	
Female	16 (32.65)	12 (75.00)	4 (25.00)		8 (50.00)	8 (50.00)	
Smoking				0.663			0.253
No	28 (57.14)	24 (85.71)	4 (14.29)		17 (60.71)	11 (39.29)	
Yes	21 (42.86)	18 (85.71)	3 (14.29)		16 (76.19)	5 (23.81)	
TNM stage				0.243			0.280
I	0 (0.00)	0 (0.00)	0 (0.00)		0 (0.00)	0 (0.00)	
II	1 (2.04)	1 (100.00)	0 (0.00)		1 (100.00)	0 (0.00)	
III	14 (28.57)	13 (92.86)	1 (7.14)		12 (85.71)	2 (14.29)	
IVa	25 (51.02)	19 (76.00)	6 (24.00)		15 (60.00)	10 (40.00)	
IVb	9 (18.37)	9 (100.00)	0 (0.00)		5 (55.56)	4 (44.44)	
Distant metastasis				0.175			0.404
Yes	9 (18.37)	9 (100.00)	0 (0.00)		5 (55.56)	4 (44.44)	
No	40 (81.63)	33	7		28 (70.00)	12 (30.00)	
Lymph node metastasis				0.680			0.482
Yes	48 (97.96)	41 (85.42)	7 (14.58)		32 (66.67)	16 (33.33)	
No	1 (2.04)	1 (100.00)	0 (0.00)		1 (100.00)	0 (0.00)	

CTCs, Circulating Tumor Cells; n, Case Number; MCTCs, Mesenchymal and Mixed CTCs; SD, Standard Deviation; TNM, Tumor-Node-Metastasis.

### Relationship between CTC profiles and the characteristics of patients with NPC

**Figure 2:**
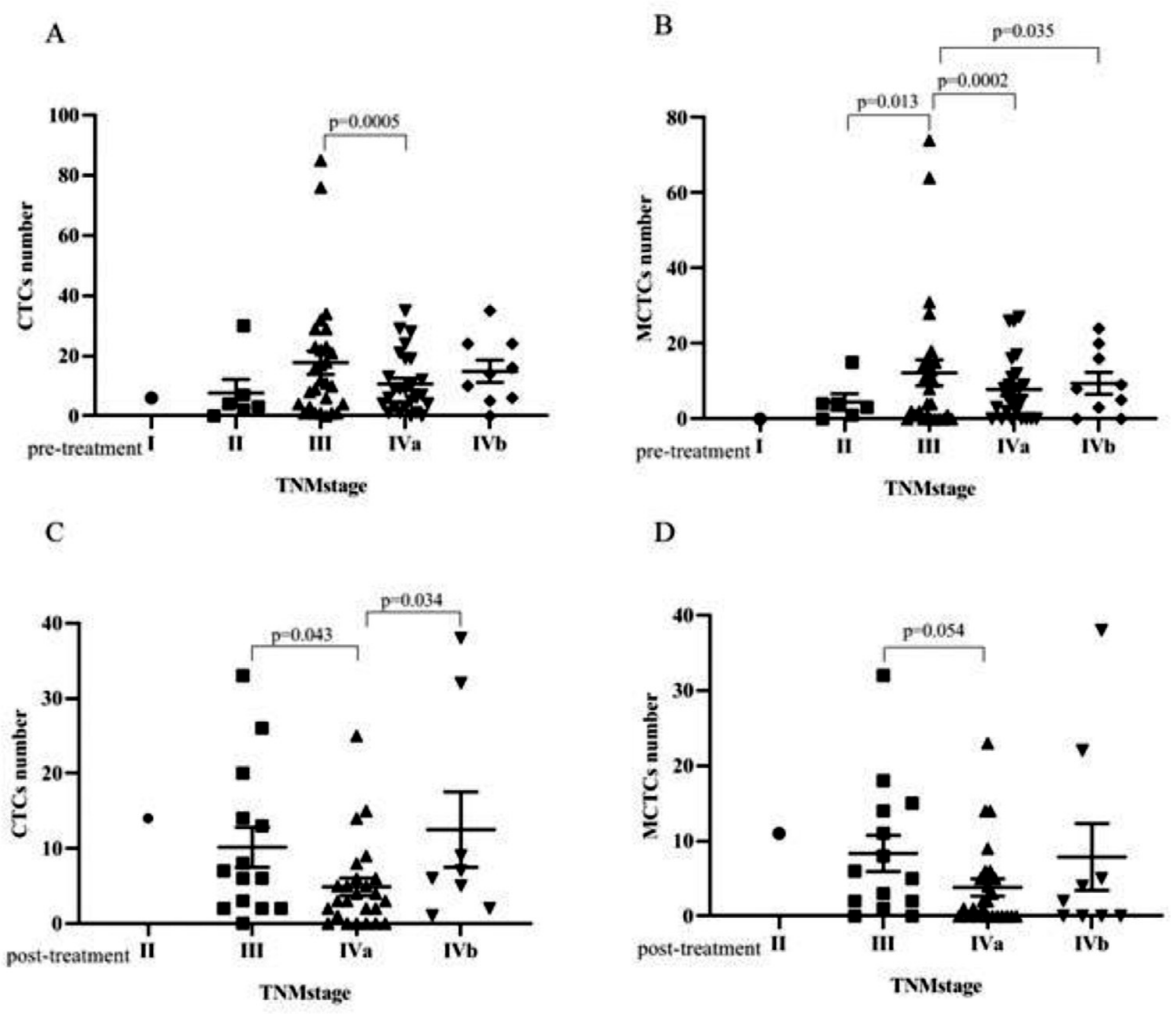
**Correlation between CTC number, MCTC number and TNM stage.** (A) In the pre-treatment group, the CTC number had significant difference between stage III and stage IVa. (B)In the pre-treatment group, the MCTC number had significant difference between early stage III and stage II, stage IVa, stage IVb. (C) In post-treatment group, CTCs number of the patients with stage IVa were statistically lower than those who with stage III and stage IVb. (D) In post-treatment group, the MCTCs number had significant difference between stage III and stage IVa*.*

Bivariate analyses of the correlation between the CTC and MCTC positivity rates and the characteristics of patients with NPC are listed in Tables 2 and 3 (pre-treatment and post-treatment groups). The results showed that CTC or MCTC positivity rates had no association with patient characteristics (p > 0.05, in all cases; Tables 2 and 3). However, the authors found that the total number of CTCs and MCTCs was associated with the TNM stage (I, II, III, Iva, IVb) (p < 0.05, Fig. 2). In both groups, patients at stage III had the highest numbers of CTCs and MCTCs statistically, and the authors hypothesized that total CTCs and MCTCs were related to metastasis. In the pre-treatment group, the median number of CTCs in patients with stage III tumors was 11.5, which was significantly higher than that in patients with stage II tumors, even stage Iva and IVb (median: 3.5, 8, 10) and stage I (only 1 sample, Fig. 2 A and B). Similarly, the authors found that the median number of MCTCs in patients with stage III tumors was 6. In contrast, the median MCTC number was 3.5 and 5.5 at stage II and stage IVa or Ivb, respectively. In addition, in the post-treatment group, the median CTCs were 6.5, 3, and 6 at stage III, IVa, and IVb, respectively. The median MCTC in stage III was 5.5, while the median in stage Iva and stage Ivb were both 1 (Fig. 2 C and D). These data revealed that CTC and MCTC had peak values in stage III, which may have been caused by metastasis.

### Associations between three therapies; the number of CTCs, MCTCs, and metastatic lesions; and the change in the number of CTC after treatment and survival

**Figure 3:**
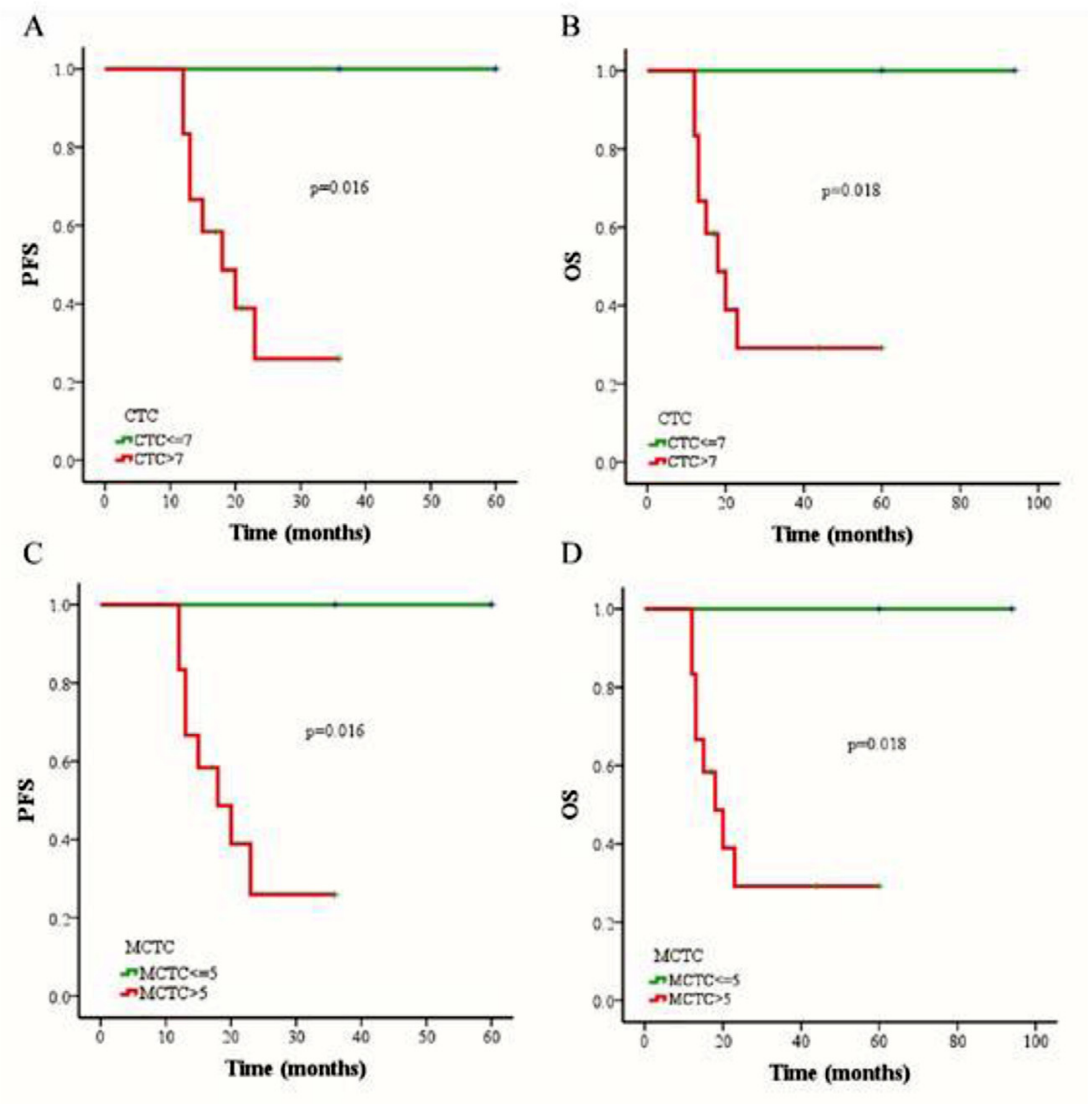
**The comparison of progression-free survival (PFS) and overall survival (OS) in NPC patients by Kaplan-Meier curves.** (A) Relationship between CTC count and PFS rate. (B) Relationship between CTC count and OS rate. (C) Relationship between MCTC number and PFS rate. (D) Relationship between MCTC number and OS rate.

**Figure 4:**
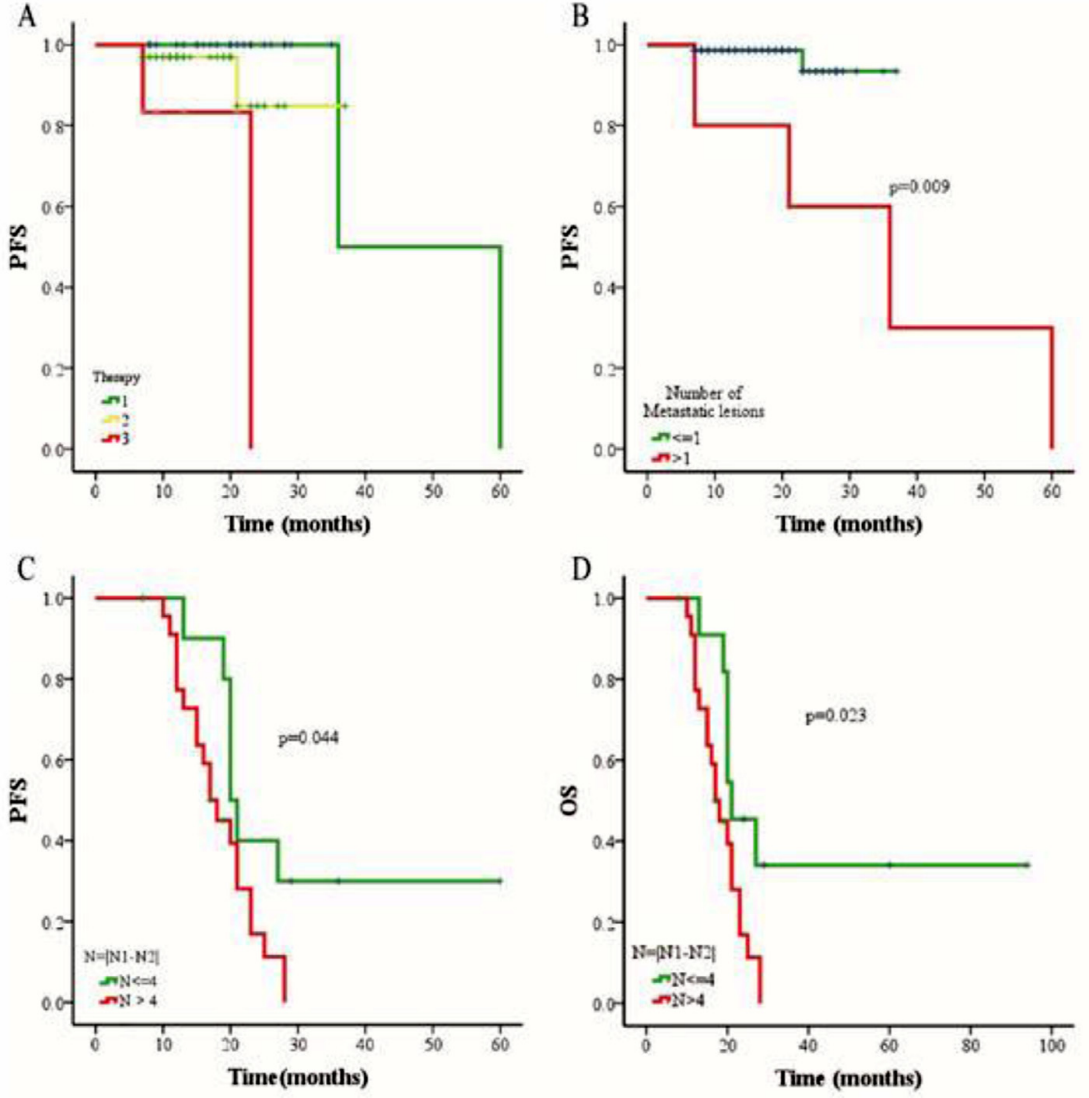
**The comparison of progression-free survival (PFS) and overall survival (OS) in NPC patients with different treatments**. (A) Relationship between three different therapies and PFS rate. Number 1 represents chemoradiotherapy, number 2 represents chemoradiotherapy combined targeted therapy, and number 3 represents chemotherapy combined targeted therapy. Statistical log rank test found that the p-value in the difference between group 1 and 2 is0.350, group 2 and group 3 is 0.065, while group 1 and group 3 is 0.0001. (B) Relationship between the number of metastatic lesions and PFS rate. (C) Relationship between the change in number of CTC after treatment and PFS rate. (D) Relationship between the change in number of CTC after treatment and OS rate. N1: the CTC counts before treatment. N2: the CTC counts after treatment. N: the absolute value of the change in number of CTC after treatment.

The outcomes of 79 patients were followed up using the RECIST assessment system. The follow-up period ranged from 7 to 94 months (median, 18 months). Among these patients, 6 (7.6%) had progressive disease and 73 (92.4%) exhibited non-progressive disease. The authors also evaluated associations among three different therapies (chemoradiotherapy, chemotherapy combined targeted therapy, or chemoradiotherapy combined targeted therapy), CTC status, CTC amount, and changes in CTC numbers of pre- and post-treatment. The authors selected a cutoff of 7 CTCs in 5 mL blood based on the Receiver Operating Characteristic (ROC) curve, and the PFS and OS rates were determined using the Kaplan-Meier method. The authors found that the PFS of patients with > 7 CTCs or > 5 MCTCs in 5 mL blood was significantly shorter than that of patients with ≤ 7 CTCs or ≤5 MCTCs (p = 0.016 and p = 0.016, respectively; Fig. 3 A and C). The presence of > 7 CTCs or > 5 MCTCs per 5 mL blood was associated with a poorer OS than ≤ 7 CTCs or ≤5 MCTCs (p = 0.018 and p = 0.018, respectively; Fig. 3 B and D). Moreover, the PFS and OS rates of patients treated with chemoradiotherapy were also significantly longer than those of patients treated with targeted therapy combined with chemotherapy or chemoradiotherapy (Fig. 4A). These results revealed that chemoradiotherapy was more effective than combined targeted therapy and chemotherapy. Moreover, the number of metastatic lesions was also associated with progression-free survival, and patients with > 1 metastatic lesion had a markedly shorter PFS than those with ≤1 metastatic lesion (Fig. 4B). Notably, analyzing the paired data obtained from 36 patients in whom the number and phenotype of CTCs were detected before and after treatment, it was found that the absolute value of the differences between the two sets of data was a potential prognostic factor for NPC. The authors investigated the prognostic potential of the changes in the number of CTCs after treatment using Kaplan-Meier survival analyses for PFS and OS. As shown in Figure 4, patients with ≥ 4 change in CTC number after treatment had a shorter PFS and OS than those with < 4 change in CTC number changed after treatment (Fig. 4 C and D).

## Discussion

Here, the authors employed the CanPatrol™ CTC-enrichment assay to isolate and characterize CTC profiles in patients with NPC. The authors first found that the total CTCs and MCTCs in patients with NPC before treatment were significantly associated with TNM staging, PFS, and OS. The authors also found that the prognosis of the patients who received chemoradiotherapy was poorer than that of patients with targeted therapy combined with chemotherapy. These results revealed that CTC count in patients with NPS is a sensitive and reliable biomarker for predicting the prognosis of patients.

Advances in techniques such as CanPatrol™ CTC enrichment have promoted the application of CTC phenotypes in auxiliary diagnosis.^28^^,^^29^ Recent studies have reported that CTCs are strongly related to the progression, relapse, and metastasis of some cancers.^30^, ^31^, ^32^ CTCs were also selected as biomarkers for patients with cancer without symptoms by the American Society of Clinical Oncology (ASCO) in 2007.^33^^,^^34^ Li et al showed that CanPatrol^TM^ detection technology can measure CTCs expression with 81.6% sensitivity and 86.8 specificities at 0.5 CTCs/5 mL cut-off in patients with non-small cell lung cancer.^14^ Qi et al. got 80.9 % sensitivity and 92.3% specificity at 16 CTC cut-offs in patients with Hepatocellular Carcinoma (HCC).^25^ Thus, CTC can be used as a useful biomarker for the early diagnosis of tumors. Here, the authors evaluated the clinical relationship between CTC count and phenotype in patients with NPC.

The present data showed that the total number of CTCs and MCTCs decreased post-treatment compared with pre-treatment. Meanwhile, the authors also found that the numbers of CTCs and MCTCs in patients with stage III tumors were significantly higher than those of patients with stage II, IVa, and Ivb tumors. Further research demonstrated that patients with > 7 CTCs or 5 MCTCs in 5 mL blood had poorer PFS and OS than patients with ≤ 7 CTCs or ≤ 5 MCTCs. In contrast, CTC ≥ 16 in HCC patients and CTC ≥ 6 in patients with pancreatic cancer were significantly prone to recurrence and metastasis.^25^^,^^35^ These data indicate that total CTCs and MCTCs are a useful biomarker for monitoring the progress of NPC patients.

Radiotherapy was recommended as the first selective treatment for patients diagnosed with advanced NPC.^36^ However, the 5-year OS rate of patients treated with radiotherapy only was 30%‒50%.^37^ Meanwhile, increasing research on chemical compounds such as SSRP1, which can reduce the proliferation of NPC tumor cells, provides new options for more effective treatment.^38^ The authors found that the combination of chemotherapy and radiotherapy was associated with significant OS rates compared to monotherapy in patients with NPC. Furthermore, the number of metastatic lesions and the changes in the number of CTCs after treatment potentially correlated with PFS or OS.

CanPatrol^TM^ detection method is a very powerful technique for specific gene expression. However, the current experiment needs multiple steps for a long time and many specific types of equipment. In addition, probes for interest gene expression need to be designed by experts. These issues may limit its extensive applications.

## Conclusion

CTC and MCTC number detection in a patient with NPC is a robust biomarker for predicting patient progress. Patients with more than 7 CTCs or 5 MCTCs in 5 mL blood had poor PFS and OS. At the same time, the number of CTCs and MCTCs was also strongly relevant to the patient's therapy.

## Authors’ contributions

Tinghua Gao and Shuping Li conceived the project and designed the experiment. Tinghua Gao, Jinxing Mao, Jindu Huang, Fengling Luo, Lixiang Lin, Yingni Lian, Sanmei Bin, Lianghua Zhao performed the experiments, collected, and analyzed the data. Tinghua Gao, Jinxing Mao and Shuping Li wrote the manuscript. All authors reviewed and approved the final manuscript.

## Funding

This research did not receive any grant support from funding agencies in the public, commercial, and not-for-profit sectors.

## Ethics approval and consent to participate

All human thyroid cancer samples were approved by the ethical committees of the First People's Hospital of Zhaoqing City (Approval#:2028-11-01). Written inform consent was obtained from all patients.

## Availability of data

All data will be available from the corresponding author upon request.

## Conflicts of interest

The authors declare no conflicts of interest.
